# Anxiety About the Risk of Death of Their Patients in Health Professionals in Spain: Analysis at the Peak of the COVID-19 Pandemic

**DOI:** 10.3390/ijerph17165938

**Published:** 2020-08-15

**Authors:** Cristina Lázaro-Pérez, Jose Ángel Martínez-López, José Gómez-Galán, Eloy López-Meneses

**Affiliations:** 1 Department of Sociology, University of Murcia, Campus Universitario, 11, 30100 Murcia, Spain; cristina.lazaro2@um.es; 2 Department of Social Work and Social Services, University of Murcia, Avda. Teniente Flomesta, 5, 30003 Murcia, Spain; jaml@um.es; 3 Department of Education, University of Extremadura, Avda. de Elvas, s/n, 06006 Badajoz, Spain; 4 College of Education, Ana G. Méndez University, Cupey Campus, San Juan, PR 00926, USA; 5 Department of Education and Social Psychology, Faculty of Social Sciences, University Pablo de Olavide, 41013 Seville, Spain; elopmen@upo.es; 6 Research Institute in Social Sciences and Education, Vice-Rectory for Research and Postgraduate, University of Atacama, Copiapó 1530000, Chile

**Keywords:** COVID-19, healthcare professionals, anxiety, death, burnout

## Abstract

The COVID-19 health crisis has had a global effect, but the consequences in the different countries affected have been very different. In Spain, in a short period of time, health professionals went from a situation of stability to living with a working environment characterized by overcrowded hospitals, lack of individual protection equipment, non-existent or contradictory work protocols, as well as an unknown increase in mortality. Although in their professional activity health workers are closely linked to death processes, in recent months, working conditions and health emergencies have drawn an unheard of working scenario, with the stress and anxiety they may suffer when faced with the death of their patients. The present quantitative research was carried out in different hospitals in Spain on health professionals during the month of April 2020. Through the subscale of anxiety in the face of the death of others, developed by Collett–Lester, it has been verified that health professionals have had to develop their work in a context of precariousness, putting at risk both their individual and collective health, notably increasing anxiety in the face of the death of their patients. The predictive variables of this anxiety have been the absence of individual protection equipment, as well as high levels in the burnout subscales of emotional exhaustion and depersonalization.

## 1. Introduction

The COVID-19 pandemic, which in recent months has attracted worldwide attention because of the large numbers of people affected and of victims, is an unprecedented event in the 21st century. It has posed a challenge to all governments and health institutions because of the impact and consequences in different areas of life such as health, political, economic, social and health management [1,2,3,4,5].

In Spain, 252,130 confirmed cases of infection, 28,392 deaths and 150,376 cured have been diagnosed by the Coordination Center for Health Alerts and Emergencies (Centro de Coordinación de Alertas y Emergencias Sanitarias, CCAES). This figure continues to rise, although now with less intensity. According to the Daily Mortality Monitoring System (Sistema de Monitorización de la Mortalidad Diaria, MoMo) [6], between 13 March and 22 May 2020, 44,546 people died in excess of the official death toll provided by the Ministry of Health.

A research by Liu et al. [7] conducted in February 2020 in Wuhan, the epicentre of the outbreak of this virus, has already shown that the main transmission routes of SARS-CoV-2, the coronavirus that causes COVID-19 disease [8], are respiratory droplets and close contact. This made healthcare workers a high-risk population especially in the early stages when the virus was not yet known and adequate protection needs for professionals had not been established.

In Spain, the Ministry of Health has defined three levels of risk faced by health professionals in relation to COVID-19 [9]. The first, “risk exposure”, includes all healthcare and non-healthcare personnel who attend to a confirmed case or a symptomatic investigation, including ambulance drivers and transport crew. The second is “low-risk exposure”, when the work activity does not include close contact with the confirmed case, such as warders, transport escorts, stretcher bearers and laboratory staff responsible for virological diagnostic tests. Finally, “low probability of exposure” includes administrative staff, medical transport technicians with a collective barrier and without direct contact with the patient, public transport drivers, security personnel, police, customs personnel, firemen and rescue workers.

These risk levels have caused many professionals to feel uneasy, especially when the number of infected healthcare workers is 52,500, representing 21% of the total number of infected people in Spain, the highest rate of infected healthcare workers in the world, and 10% of the healthcare population, double the general population [10]. This figure grew by 40% during May, according to reports from the College Medical Organization (Organización Médico Colegial, OMC), an organization that groups together all the medical associations in Spain [11].

This discomfort experienced by health workers during the first months of the pandemic was picked up by various media outlets echoing the demands of these workers: A significant risk factor faced by health professionals is that two months after the onset of the State of Alarm, workers in health centers and hospitals were still demanding two important things, massive PCR and serological tests, as these are a critical factor, and personal protective equipment (PPE) that complies with regulations [12].

With regard to the first of these, the Hospital Care Department of the Association of Physicians and Graduates of Madrid (Atención Hospitalaria de la Asociación de Médicos y Titulados Superiores de Madrid, AMYTS) reported that there was an urgent need to know whether or not the health workers were infected, whether they had passed the infection or had acquired immunity, as well as to carry out mass PCR tests [13]. The Spanish Society of Primary Care Physicians (Sociedad Española de Médicos de Atención Primaria, Semergen) reported that personal protective equipment was scarce and that there were still shortcomings [14].

This complaint, together with the fact that 58,000 units that were part of a purchase of 640,000 rapid coronavirus detection tests had to be returned to China, the country from which they originated, has generated confusion among professionals who have faced the pandemic with personal protection equipment that does not comply with regulations, such as suits made from garbage bags or face screens made from plastic files [15,16]. In many places, the first aid came from donations from companies, individuals and crowdfunding among citizens to supply the material they needed and perhaps most disturbing was the recycling and reuse of masks and gowns in up to three shifts, material that should instead be used only once [17].

The first shipments of material supplied to the toilets by the Ministry of Health also had to be returned in large part. “Garry Galaxy” (Garry Galaxy Biotechnology Co., Ltd., New York, NY, USA) masks did not comply with European regulations, and more than 1000 healthcare providers in Spain were forced to isolate them. Later, the same thing happened with two lots of the Chinese brand “Purvigor” (GuanDong Fei Fan Mstar Technology, Ltd., Foshan, China), in Andalusia and Madrid, which were removed from several hospitals and centers for the elderly [11,18].

The provision of personal protective equipment, such as masks, gloves, screens, etc., is part of the mandatory protection to which any worker must have access during his working day. These are elements of protection against risks that may threaten the safety or health of workers, according to Law 31/95 of 8 November on the Prevention of Occupational Risks [19]. However, health professionals have been unprotected against these risks during the most virulent weeks of the pandemic.

Both external factors related to the scope of the pandemic, such as the scarce information on COVID-19 and its intensity, and internal factors, such as the lack of individual protection, the lack of foresight and the establishment of adequate protocols, have been a source that has generated and sustained occupational stress among health center workers, hospitals and nursing homes, since in addition to facing the virus itself and its consequences—illness, isolation, extreme prevention in contact with health workers’ families, death, in some cases even of infected colleagues at their workplace—they do not feel protected by the competent national authorities. The paradox is that, on the one hand, the Spanish Government was designated as the sole Competent Authority in the State during the alarm decree, putting health professionals at the head of COVID-19 but without guaranteeing their individual health as they could be a mechanism for transmitting the disease, thus also putting collective health at risk.

The dizzying increase in the number of infections has increased the workload in a very short period of time, and with it the probability of infection among professionals, as well as the fear of infecting the relatives with whom they live, so that many of these professionals who dealt daily with the virus had to isolate themselves in their own homes or set up a new residence in the hotels fitted out for the health workers who needed a room. A study in Ireland found that in this context of COVID-19, health workers with children experience greater psychological distress because of this mentioned possibility of transmission to family members [20]. At the same time, many of them have seen their jobs endangered in recent weeks, when the number of infections has been decreasing, which has generated more stress than the existing one, in addition to anxiety and depression due to the intense circumstances experienced and generated by the appearance of COVID-19 [21].

One aspect that should be taken into account during the pandemic, in addition to physical health, is the mental health of health professionals. Several authors consider that such care would be very important in helping to control the disease [22,23,24]. Along these lines, identifying the high-risk healthcare population by contagion, as well as those who require adequate psychological care, emerges as a preferential action by healthcare institutions to provide the emotional balance needed by their workers, especially considering that the fight against this disease is not yet over, and new outbreaks are constant throughout the world.

Health professionals are directly involved with patients with COVID-19 in different phases: their diagnosis, treatment and care, and even in the death process, given that family members could not accompany patients with COVID-19 who died alone. Because of this, they are more vulnerable to psychological disorders such as stress, depression and anxiety [25]. In studies prior to the outbreak of COVID-19, almost 50% of health professionals suffered from work-related stress and exhaustion [26]. Following the emergence of the pandemic, mental health problems among health workers have become more evident and have been highlighted.

In China, on 3 February 2020, the National Health Commission (NHC) issued a statement calling on regions at the provincial level to integrate mental health support resources and standardize such public services. Wang Bin, deputy director of the NHC’s disease prevention and control office, urged local governments to coordinate a variety of mental health hotlines provided by education authorities, civil affairs departments, or social organizations, while offering training and guidance to hotline workers. The NHC issued a protocol for emergency mental intervention during the new coronavirus outbreak. The guideline proposed a four-level system based on the risk of developing mental problems: patients confirmed to have the virus, medical personnel on the front line, disease prevention and, finally, management personnel placed at the highest level [27]. In addition, on 15 February, the importance of providing psychological intervention and support among health professionals was again communicated [28].

In Spain, psychological assistance to both healthcare personnel and the general population has been offered free of charge through the devices that the official associations of psychologists and various associations throughout the country have implemented as a solidarity contribution in the face of the overwhelming situation caused by the COVID-19 pandemic [29]. However, these actions do not form part of a national strategy with homogeneous and standardized guidelines for the treatment of health professionals in need of psychological treatment. Nor have the antecedents and proposals made by the NHC in China been helpful.

On the other hand, on 22 March, a press release was issued by the Official College of Psychologists (Colegio Oficial de Psicólogos) of Madrid, the autonomous community with the most affected people in Spain, where it was announced that in collaboration with different professional societies and colleges, the health services of the Community of Madrid would be attended to in situations of high levels of stress, overload of work and emotional demand, which could have a very negative effect on their psychological health. Support would begin progressively, first to the internists, emergency care providers and also to professionals working in nursing homes and would be extended to other health groups [30].

A little over a month later, and after nearly 10,000 interventions, on April 29 they issued a statement announcing the imminent cessation of this service due to the impossibility of maintaining the resource in the conditions in which it was being developed, setting May 3 as the end date for their collaboration [31], the date on which Spain was still in a State of Alarm.

This lack of provision of psychological resources in crisis or overflow situations in general, and in daily practice in particular, makes evident the lack of recognition of the importance of emotional management in the healthcare setting. The recent literature related to this topic shows the relevance of this type of care [32]. Along the same lines, in regions of China most affected by the COVID-19 pandemic, a 7% incidence of post-traumatic stress symptoms was reported one month after the outbreak of the pandemic [33], evidencing the need for psychological care and prevention in emergency situations not only during the months of greatest infection but also afterwards.

In this health crisis, the importance of two aspects that stand out in relation to the psychological and emotional care of health professionals and that can interfere with their therapeutic work has been highlighted. It has been relevant to reflect on them in relation to all the above: work stress and anxiety or fear of death, especially bearing in mind the process of dying of patients, which are the lives they have tried to save and the deaths they have had to face daily during the onset of the pandemic.

## 2. Background

Anxiety is a set of physical, mental and motor manifestations not attributable to real danger, but arising either suddenly or as a constant, imprecise state [34,35]. Although it shares with fear sensations of apprehension, danger and physiological reactions, anxiety is not linked to present stimuli, as in fear, but to possible future dangers, which are neither definable nor foreseeable [36].

Death has been studied extensively in different societies and cultures [37,38,39,40,41]. The enigma of the possible transcendence of consciousness or of something of unknown origin that survives us has been discovered in order to give meaning to human existence and above all to avoid suffering in the face of the unknown and fear of the finiteness of life, which causes extraordinary uneasiness, especially in the face of the loss of loved ones [42]. On the other hand, and as a shield to minimize the effects that thinking about death can generate and attenuate this anxiety in the face of death, the human being is involved in developing self-esteem, provoking the feeling of being a valuable member of the culture to which he belongs and being able to be remembered after death. [43] Thus, in all spheres of life we find evidence of these attempts at permanence: literature, works of art or monuments are examples of this.

This awareness of death can produce a sensation of confusion, meaninglessness or fear and generate undesirable behavior [44]; some cases are the result of mental health problems, and others are not directly related to death but generated by this awareness of the end of existence.

Flight and avoidance are two psychological defense mechanisms that, when faced with the perception of death, human beings usually include in their behavioral repertoire. All information related to the possibility of illness or death is avoided, such as news related to the evolution of the pandemic or figures of contagion and deaths so that if there is no knowledge, it is as if the problem did not exist. On the other hand, contact with potentially dangerous groups, people or situations is avoided, which can generate an isolation that protects but also alienates socially.

This fear of ceasing to exist is often manifested in human beings by two aspects that can influence the degree of anxiety that death can cause: balanced mental health and non-traumatic personal experiences as well as prior personal management of a situation close to death [45] can help to minimize the degree of anxiety in the face of death, stress or psychological distress and in situations of great impact, dealing with the situation in such a way that reactions are more adaptive.

This ideal way of dealing with stressful processes that can cause anxiety is very present in the health professions due to prolonged exposure to death [46,47,48,49] and is a generator of work-related stress or burnout [7,50,51,52,53,54] that is associated with monotonous tasks or excessive burden, the amount of time that the healthcare provider deals with patients, poorer self-rated health and prolonged absences due to illness [20,55]. As recent studies show, certain traumatic events can diminish the sense of security of some people, evoke death and negatively influence their mental health [55], and the greatest risk of transmission of the virus is to medical carers [56].

There is relevant information in the scientific literature about the high risk that health professionals have of presenting disorders derived from their professional practice, such as high levels of exhaustion, depression, stress, anxiety [57], some type of addiction and even post-traumatic stress disorder, which could have long-term psychological consequences [58,59,60], especially in situations of health emergencies [61]. In some cases, psychological intervention by a professional may even be necessary [62,63]. It is, therefore, of paramount importance to know to what extent health professionals know and can maintain self-care [64,65] to mitigate the possible harm caused by emotional pain arising from their therapeutic work, keep good mental health, and identify factors that may cause some type of depressive or other disorders in the workplace [66]. In this way, their ability to care for patients by providing better quality of care can be protected and will remain intact.

During the COVID-19 pandemic, the psychological impact on healthcare providers was exacerbated in the workplace by interpersonal and social distancing, new infection control protocols and working in unfamiliar contexts and with unknown colleagues, while in the family setting, healthcare providers with children suffered greater psychological distress, although family support protected against work-related stress [20].

In the first half of 2020, health professionals have faced the extraordinary phenomenon of increasing mortality in a short period of time, without being able to foresee or be sure that health resources will arrive in time to have the desired effect on the cure of patients, nor being able to prevent deaths with the pharmacological and life support treatments administered. For this reason, the object of the present study was to find out the degree of anxiety derived from the death of their patients, since, in addition to the work of the physical care inherent in their therapeutic task, they had sufficient empathy to accompany the patients so that they did not die alone, a task traditionally carried out in Spain by the relatives.

Fear or anxiety in the face of death is deduced from the behavior, reactions and responses of the individual, since it does not manifest itself directly. For this reason and because of the absence of instruments to measure attitudes towards death, the authors Collett and Lester [67] created the Fear of Death scale, highlighting the multicausal component of this fear, whose attitudes and reactions depend on whether anxiety is a state or a trait [68,69]. These authors distinguished 4 subscales: fear of one’s own death, fear of the death of others, fear of the process of one’s own death and fear of the process of death of others [67,70].

The extraordinary nature of the pandemic and the new procedures put in place to try to minimize the impact of SARS-CoV-2 meant that health professionals had to make a great effort, which as in previous situations of maximum health stress, led to anxiety crises and anxiolytic intake [71] with the intention of maintaining the quality of care of their patients. This continuous attention to the aforementioned stressors, which are the result of this unusual health crisis, can lead to depressive episodes and post-traumatic stress [32,72].

Anxiety about the death processes of patients is determined by the high levels of burnout suffered by health professionals [26,54,73,74,75]. Burnout is present in many professions, especially those linked to death processes and is considered to be the prolonged response to chronic stress at a personal and relational level at work, determined from the dimensions known as exhaustion, depersonalization and professional cynicism and inefficiency [76].

Maslach and Jackson [77] developed a scale to analyze high levels of burnout composed of three subscales: Emotional Exhaustion (EE), Depersonalization (DP) and Personal Accomplishment (PA). Emotional burnout is determined by gradual loss of energy and exhaustion; that is, it is the subjective perception of feeling emotionally exhausted by the demands of the job. In relation to depersonalization, this subscale values the personal detachment that workers suffer leading to negative attitudes, even to the point of blaming patients for problems that happen to them as workers. Thirdly, the lack of Personal Accomplishment is characterized by negative responses to the worker himself, as well as in relation to their work, even with pseudo-depressive manifestations, tendencies to flighting, physical and psychic exhaustion and dehumanization.

In the health crisis caused by COVID-19, these factors may have been aggravated by the virulence of the pandemic and the scarcity of adequate means of protection, which makes it interesting to know how health professionals have faced this public health crisis, especially during the most critical weeks of the spread of the virus.

## 3. Methods

### 3.1. Objectives

The aim of this research is twofold: (a) to find out whether anxiety has been produced on the part of healthcare workers in relation to the processes of death of their patients and (b) what variables are involved at this level. The methodology developed has been eminently quantitative during the weeks of greatest virulence of the virus through the preparation of a questionnaire designed ad hoc from the Collett–Lester death anxiety scale [67], translating it into Spanish and, obviously, applying it in Spain.

### 3.2. Study Design

The research has been carried out in different hospitals in Spain to health personnel between the second and third weeks of April. During these two weeks, Spain was in a period of confinement of the population as a result of the risk of spread of the virus, and the figures of mortality and infections reached the highest levels during the pandemic. A total of 157 questionnaires were completed in different hospitals in 12 Autonomous Communities in Spain. The design of the investigation was through a simple non-probabilistic random sampling. There are several reasons for this design: (a) the need to obtain data at a historical moment, (b) the reduced time space for collecting these data and (c) the difficulties of participation by health professionals before the harsh working conditions in which they found themselves.

It is worth mentioning the enormous difficulty we had in accessing health professionals both as a result of the State of Alarm (which implied absolute confinement) and the extreme conditions in which they were doing their work. In spite of this, we were able to obtain a sample of dozens of health professionals, which has allowed us to obtain valuable information about one of the most exceptional moments, without a doubt, that have occurred in Spain since the Civil War.

As a result of the declaration of the State of Alarm, the confinement of the population, the low mobility of citizens and the pressure to which health professionals were subjected by the increase in the mortality rate of their patients, access to participants was made through health professional associations/entities working in hospital centers. Due to the nature of the research and the need to obtain immediate data on the effects of the pandemic, this model was chosen. The instrument administered was an online survey through a virtual tool available at the University of Murcia, which allows for rapid distribution, analysis and exploitation of data. This was a determining factor in reaching such a high sample in a short time during the most virulent weeks of SARS-CoV-2 in Spain. Therefore, although from a methodological point of view it would have been advisable to reach a larger number of participants, we assumed the sample reached was acceptable since it was necessary to analyze this social and health phenomenon immediately in order to improve both the care given by health professionals to their patients and the approach to disease prevention and anxiety in the health workers themselves.

The protocol for the field work followed all the guidelines of the Ethics Committee of the universities to which the members of the research team belong. As this is a descriptive study in Spain, official approval by the committees is not necessary (it should only occur in the case of experimental studies). However, all the Codes of Good Practice for Research on Human Beings were followed punctually. The study process, from project to conclusion, was signed and registered (code Nº REPRIN-PEM-03) by the research team that comprised the authors. It should also be noted that all participants gave their informed consent in accordance with the Declaration of Helsinki. The instrument used to collect the information, in the form of an online questionnaire that fully guaranteed confidentiality and anonymity, implies acceptance of the conditions for access to it. Participants must accept the ethical conditions and give their consent before accessing the questionnaire and submitting their responses.

The questionnaire consisted of 17 items divided into three sections: (a) sociodemographic, related to the level of anxiety in the face of the death of others (patients), and (b) related to characteristics of the working environment. From a socio-demographic perspective, the following items were included: (1) sex, (2) age, (3) professional category and (4) Autonomous Community. In relation to the scale of anxiety in the face of the death of others, the items from the Collett–Lester scale [67] were included: (5) having to be with someone who is dying, (6) having them want to talk about death with you, (7) watching them suffer from pain, (8) having to be the one to tell them that they are dying, (9) seeing the physical degeneration of their body, (10) not knowing what to do about your grief at losing them when you are with them, (11) watching the deterioration of their mental abilities, (12) being reminded that you are going to do through the experience also one day. Finally, the questionnaire included the following items related to the work environment: (13) BMI level (Maslach and Jackson’s scale) through its 3 subscales: Emotional Exhaustion (EE), Depersonalization (DP) and Personal Accomplishment (PA), (14) need for psychological treatment, (15) need to incorporate psychological/psychiatric treatment, (16) personal need for future psychological treatment and (17) assessment of how the lack of PPEs (Personal Protection Equipment) may be affecting their level of stress or anxiety.

### 3.3. Variables Used

#### 3.3.1. Dependent Variable

The Collett–Lester Fear of Death Scale [67] was used to measure patients’ anxiety about death. This scale is made up of 4 subscales which provide information from a multidimensional perspective on “Fear of one’s own death”, “Fear of the process of dying one’s own death”, “Fear of the death of others” and “Fear of the process of dying of others”. In our research, we focus on this last subscale: “Fear of the process of dying of others” referred in this study to the death of their patients. The object of the study is anxiety about the processes of dying of others, in this case, of patients by health professionals. For this reason, and understanding the context of personal, family and work difficulties of these workers, it was determined appropriate to use only Subscale 4, anxiety in the face of the processes of death of Collett–Lester [67]. The response options are distributed on a Likert-type scale from 1 (nothing) to 5 (a lot). This subscale is composed of 7 items, and its final value is averaged over the response set. Thus, the higher the average of the responses, the greater the fear of the death processes of others, in this case, the patients with whom they worked. This variable was transformed into a dichotomy differentiating between low (lowest possible level to average level) and high (from average to highest possible level) levels of suffering anxiety as a consequence of the death processes of others.

#### 3.3.2. Independent Variables

Three types of independent variables were established: (a) socio-demographic, (b) subjective perceptions of the current situation at work and (c) the Maslach Burnout Inventory (MBI) subscales [77]. The following are used for sociodemographic variables: Sex (female/male), Age (continuous) and Professional Category (Nurse/Assistant Nurse, Doctor, Other). The following variables were used for the subjective variables: need for psychological treatment (Yes/No), need to incorporate psychological/psychiatric treatment (Yes/No), assessment of personal need for future psychological treatment (Yes/No), and assessment of how the lack of PPEs (Personal Protection Equipment) may be affecting their level of stress or anxiety (Yes/No). Finally, in relation to BMI, the three subscales Emotional Exhaustion (EE), Depersonalization (DP) and Personal Accomplishment (PA) were used. These variables were established dichotomously based on low or medium/high values.

### 3.4. Statistical Analysis

The statistical analysis was carried out in 3 stages through the statistical program SPSS Statistics 24 (IBM Corp., Armonk, NY, United States). Firstly, a frequency analysis of the set of variables was carried out in order to make a first approximation to the phenomenon under study. Subsequently, to observe the relationships between the variables, a cross-table analysis was performed, taking into account the significance of chi-square *p* < 0.005. Finally, a logistic regression was carried out to know the probability of occurrence (risk of anxiety before the processes of death of patients) from the independent variables.

## 4. Results

In the first place, the majority of participants in the study were women (79.0%), with a low representation of men (21.0%). These data are in line with the feminization of the labor market in relation to health services [78]. Three age subgroups were classified according to age. The most numerous corresponded to those under 41 years of age, which represented 47.8%. Secondly, 42.0% of those surveyed were between 41 and 60 years of age. Finally, persons aged >60 years old accounted for 10.2%.

With regard to the professional category, Nurse/Assitant Nurse stood out with 69.4%. Subsequently, we found doctors with 14.0% and others with 16.6%. Regarding whether the lack of PPEs causes them stress and anxiety, 85.4% answered yes. The following results were obtained for the burnout subscales: 58.6% showed low levels of emotional exhaustion and 41.4% medium/high levels. In the rest of the subscales, higher levels were obtained. In the case of Depersonalization, the low level reached 31.8%, while 68.2% presented medium/high levels. Finally, in relation to personal fulfilment, 45.9% have low levels while 54.1% show medium/high levels. In the specific application of the scale of anxiety about the death of others, 28.7% of the persons surveyed had a low level and 71.3% had a high level. It is presented in Table 1.

Looking at the analysis of the crossed tables, we can see that the dependent variable, high levels of anxiety before the processes of death of others, is associated with the following independent variables attending to a level of significance of the chi-square *p* < 0.05. Firstly, the association between the dependent variable and sex (0.042) stands out. Secondly, we observe how this variable is also associated with the subjective perception of whether they consider that psychological support should be provided in the work centers (0.032). The association of the dependent variable with the subjective perception of whether the lack of PPE can produce a high level of anxiety in the face of the death of others is almost perfect (0.001). In addition, the association with the three burnout subscales stands out: Emotional Exhaustion (0.033), Depersonalization (0.007) and Personal Accomplishment (0.040). Therefore, it can be seen that the burnout levels in health professionals are closely related to the high levels of anxiety of these professionals in the face of the death processes of their patients.

Finally, with the aim of predicting the probability of the event, binary logistic regression was performed using the Forward method to find out which independent variables could determine the probability of having a high level of anxiety when faced with the death processes of patients by healthcare professionals. The variables used in the logistic regression are show at the Table 2.

The logistic regression model was statistically significant, X^2^ = 24.100, *p* < 0.005. The model explains 22.4% (Nagelkerke’s R^2^) of the variance in the risk of death anxiety in the death processes of others and correctly classifies 81.5% of the cases. The Hosmer–Lemeshow test showed that there were no significant differences between observed and predicted results in the model with a *p* = 0.454.

As for the variables predicting the event, the following were significant: (a) The absence of PPE generates increased levels of stress and anxiety, (b) Emotional Exhaustion and (c) Depersonalization.

The main results are shown in Table 3. In the specific case of subjective perception of whether the lack of PPE presents an Odds Ratio (OR) = 4.021, IC 95% (1.479 to 10.932), *p* = 0.006. It is striking that health professionals who have occupational anxiety as a result of the lack of PPE are up to 4 times more likely to suffer anxiety when faced with the death processes of their patients. The OR for EE was 2.997, 95% CI (1.105 to 8.124), *p* = 0.031. DP presented an OR = 3.096, IC 95% (1.313 to 7.303), *p* = 0.010. Burnout variables according to the subscales of Emotional Exhaustion and Depersonalization are explanatory in the increase of anxiety of health professionals. In fact, in both cases, the risk of suffering anxiety in the face of the death processes of patients increases by 3 points if there are moderate/high levels of Emotional Exhaustion and Depersonalization.

## 5. Discussion

If the current pandemic resulting from Covid-19 has taught us anything, it is that we need to analyze the work context where health professionals exercise their profession, especially in those situations linked to the processes of death of patients. Although death continues to have taboo connotations for much of society [79], the same is not true for health professionals, whose proximity to these processes means this short life cycle is understood as just another stage in the humanization of care. Until very recently, a large part of the medical community understood death as the failure of medicine, ignoring in some cases palliative care in favor of therapeutic fierceness, so that the patient was not allowed the natural acceptance of their situation, and the family were given false hope of recovery by interfering in their grieving process [80].

However, we cannot put in perspective the impact that the death processes of their patients may have on health professionals, much less the events that took place during the current health crisis in Spain, where on occasion there was not even space to store the bodies and coffins, as occurred in Madrid where they had to fit out the Ice Palace (Palacio del Hielo) for this purpose.

The present research has deepened on a known and studied phenomenon, the anxiety before the death, contextualized in the hardest weeks of the sanitary crisis of the COVID-19 in Spain. Taking into account the objectives set out, it has been verified that the majority of health professionals showed very high levels of anxiety in the face of the death of their patients during the period of implementation of the research, reaching high levels in 79.4% of health workers.

On the other hand, we observe that the variables that have most conditioned this anxiety are linked to the lack of PPE and high levels of burnout, highlighting emotional exhaustion and depersonalization on this scale. These variables are predictive of the phenomenon of having high anxiety in the face of the death of others.

Anxiety and stress regarding the process of dying of others in health professionals during this temporary period of high mortality is in turn conditioned by high levels of burnout [81]. This connection between high levels of burnout and anxiety or stress in health professionals is a scientific reality that has been confirmed by numerous studies over the past few decades [47,48,49,73,74,75,82,83,84,85]. These results point to consequences and implications for the professional work of these workers, since we do not have objective data showing a change in the trend or a lower risk of outbreaks. As observed in the results of the present study, the mental health of health workers is vulnerable to events related to their patients; they present important levels of stress and anxiety in the face of the death of the people they care for as previous research has shown [57,58,59,60,66], although in the context of the COVID-19 health crisis, it is more evident because of the short period of time in which deaths have increased significantly. However, we cannot forget the effects that the lack of PPE is having on the current health crisis, as it is the greatest predictor of anxiety in the face of the death of others. This is not a causal result in the Spanish case but is connected with numerous studies being conducted worldwide [7,51,52,53,86,87,88,89,90,91].

All these elements are negatively influencing their psychological situation, increasing their vulnerability at a historical moment, just as it is happening in many countries [92,93,94,95]. Although no one could have foreseen the consequences and scope of the current pandemic, health professionals have been carrying out their work without obtaining an adequate response from government institutions and public managers, as other studies also point out [20]. Thus, anxiety and stress in the processes of death of others as a result of the effects of this pandemic should be studied to improve the mental health of health professionals.

Finally, it should be stressed that in this research, there has been a significant limitation in access to health professionals as a result of the declaration of the State of Alarm and the conditions in which they carry out their work. In spite of not having developed a stratified research in the whole of the Spanish territory, the results allow access to information of vital importance both for the current management of the health crisis and to design, plan and implement actions to improve the mental health of health professionals subjected to high levels of anxiety and stress at work due to the death of their patients.

## 6. Conclusions

The SARS-CoV-2 virus has highlighted how poorly prepared health institutions were for a pandemic like the one we are experiencing at the beginning of this century. Despite continuous scientific warnings that the time would come, the lack of coordination on the part of international agencies, as well as the public management of all the countries where the COVID-19 disease has been most virulent, has shown that there was not a sufficiently amplified alarm signal to mobilize national governments or that they did not pay enough attention, relativizing the effects of the virus, or perhaps both reasons provided the breeding ground for the spread of the virus in the global era, despite the declaration of the current outbreak of the new coronavirus as a Public Health Emergency of International Importance (PHEIC) by the International Health Regulations Emergency Committee (IHR, 2005) on 30 January 2020 [96].

In this pandemic, health professionals have had to deal with an unknown virus, with insufficient means and immersed in constant work stress, where deaths increased at an exponential rate without the ability to find safe treatments or the conviction that through healthcare they could cure the patients of COVID-19. Health professionals have suffered an increase in anxiety and stress at work, putting their lives at risk and living with death around them like never before, which may even lead to medical negligence [20].

The absence of specific protocols and effective and efficient planning by government institutions, contradictory guidelines by middle managers, as well as inadequate hospital health coordination have meant a risk not only to the mental health and well-being of health professionals themselves but also to patients, given that they carry out their work in a context of high stress and anxiety.

In this way, these health and government institutions are jointly responsible for both individual health—at the professional level of health workers—and collective health. For this reason, these results should provide scientific evidence of the need to improve several elements. On the one hand, there is health coordination at an international level. In a global world where the SARS-CoV-2 virus is circulating worldwide at a speed unprecedented in history, a globally planned strategy is needed where collaboration and health coordination emerge as principles for action. Secondly, health professionals must be adequately protected to deal with this disease. The absence of PPEs by healthcare professionals during the current pandemic in Spain has demonstrated the shortcomings not only in management but also in planning and coordination both from an international perspective and within the country itself. Finally, it is necessary to attend to and protect the mental health of health professionals who have been working directly on the death processes of others, from a double perspective: prevention and treatment. What happened in Spain with the current pandemic has exposed a lack of management and protection of health professionals that should not be repeated.

## Figures and Tables

**Table 1:** Descriptive analysis of the variables.

Variables	N	%
Gender		
Woman	124	79.0
Man	33	21.0
Age		
<41	75	47.8
41–60	66	42.0
>60	16	10.2
Work		
Doctor	22	14.0
Nurse/N.A.	109	69.4
Other	26	16.6
Absence of PPE, increases stress/anxiety		
Yes	134	85.4
No	23	14.6
Sub Emotional Exhaustion		
Low	92	58.6
Medium/High	65	41.4
Sub De-Personalization		
Low	50	31.8
Medium/High	107	68.2
Sub Personal Accomplishment		
Low	72	45.9
Medium/High	85	54.1
Anxiety about the death of others		
Low	45	28.7
High	112	71.3

**Table 2:** Variables used in binary logistic regression.

**1. Gender**
Ref. Man
(1) Woman
**2. Age (Constant)**
**3. Job**
Ref. Others
(1) Nurse/Assistant Nurse
(2) Doctor
**4. Needs Psychological/Psychiatric Support**
Ref. No
(1) Yes
**5. Psychological/Psychiatric Support Should Be Provided**
Ref. No
(1) Yes
**6. Will Need Psychological/Psychiatric Support**
Ref. No
(1) Yes
**7. Lack of PPEs Causes Anxiety and Stress**
Ref. No
(1) Yes
**8. Emotional Exhaustion**
Ref. No
(1) Yes
**9. Depersonalization**
Ref. No
(1) Yes
**10. Personal Accomplishment**
Ref. No
(1) Yes

**Table 3:** Binary logistic regression results.

	e^b^
**PPEs (1)**	4.021 ^**^
**Emotional Exhaustion (1)**	2.997 ^*^
**Depersonalization (1)**	3.096 ^*^
**Constant**	0.462

e^b^ = Exp (B). * Sig (Significance level) = 0.05; ** Sig = 0.001.
